# Rhino‐Orbital‐Cerebral Mucormycosis With Bilateral Endogenous *Aspergillus* Endophthalmitis Following Steroid Therapy for COVID‐19 Infection

**DOI:** 10.1155/crop/6610355

**Published:** 2026-06-07

**Authors:** Nasser Shoeibi, Mohammad Etezad Razavi, Farid Shekarchian, Hamid Reza Heidarzadeh, Seyedeh Maryam Hosseini

**Affiliations:** ^1^ Eye Research Center, Mashhad University of Medical Sciences, Mashhad, Iran, mums.ac.ir

**Keywords:** *Aspergillus*, coronavirus disease 2019 (COVID-19), endophthalmitis, mucormycosis

## Abstract

**Purpose:**

We report a diabetic patient with rhino‐orbital‐cerebral mucormycosis (ROCM) and bilateral endogenous Aspergillus endophthalmitis post‐coronavirus disease 2019 (COVID‐19) infection.

**Case Report:**

A 45‐year‐old man who had diabetes experienced vision loss and sought medical assistance. Prior to developing orbital symptoms, he had undergone intensive corticosteroid therapy for COVID‐19. His uncorrected visual acuity was 20/20 in the right eye, but he had no light perception in his left eye. While hospitalized for ROCM, he developed endogenous endophthalmitis in his left eye, which was caused by Aspergillus fumigatus. Additionally, Aspergillus retinitis was diagnosed in his right eye due to the development of white retinal lesions and preretinal hemorrhage. To treat ROCM, he received systemic and retrobulbar injections of amphotericin B, which led to regression. Posaconazole was prescribed for the treatment of Aspergillus endophthalmitis. The retinitis lesions in his right eye responded well to oral posaconazole without any long‐term complications.

**Conclusion:**

Patients with COVID‐19‐associated immunodeficiency may be more vulnerable to opportunistic pathogens, such as mucormycosis and Aspergillus, particularly if they have comorbidities like diabetes mellitus. It is also possible for one person to be coinfected with multiple opportunistic pathogens.

## 1. Introduction

The ongoing second wave of the COVID‐19 pandemic has led to a surge in rhino‐orbital‐cerebral mucormycosis (ROCM) cases across the globe. This fungal infection, previously identified as an opportunistic and angioinvasive disease, is now called COVID‐19‐associated ROCM. Individuals with immunodeficiency factors such as diabetes mellitus (DM) and those taking systemic corticosteroids are at higher risk of contracting this infection [1].

*Aspergillus* endophthalmitis is a condition that typically affects individuals with underlying immunodeficiency, often due to corticosteroid use or DM. The pathogenesis of this condition involves either the direct entry of the *Aspergillus* organism into the eye (exogenous) or the hematogenous spread of the organism (endogenous) following fungemia. It is important to note that the incidence of *Aspergillus* endophthalmitis is relatively rare, but it can be a serious complication for individuals with compromised immune systems [2].

This report describes a patient with a history of DM and COVID‐19 who received intensive corticosteroid treatment and presented with ROCM, which progressed to bilateral *Aspergillus* endophthalmitis.

## 2. Case Report

A 45‐year‐old male with ROCM was referred to the emergency department. He had a history of DM and moderate COVID‐19 (positive real‐time reverse transcription‐polymerase chain reaction result for severe acute respiratory syndrome coronavirus‐2 (SARS‐CoV‐2) in a nasopharyngeal sample with evidence of lower respiratory involvement in imaging and SpO_2_ ≥ 94*%* on room air), which was treated out‐patiently with systemic corticosteroid (dexamethasone 8 mg twice daily for 10 days) and supportive therapy. He did not receive remdesivir. Fifteen days before presenting initial orbital symptoms, he had finished receiving dexamethasone. He developed gradual chemosis and proptosis of the left eye, and his vision decreased 2 days later. He had a history of four‐time endoscopic debridement of the left‐sided nasal cavity and perinasal sinuses with confirmed pathology specimens of mucormycosis. Surgical debridement included removal of the left lamina papyracea with ethmoidectomy and sphenoidectomy, along with resection of the left maxillary sinus ostium. He was receiving intravenous liposomal amphotericin B.

The ophthalmology service was consulted on the 12th day after admission. The patient had a normal uncorrected visual acuity (UCVA) of 20/20 in the right eye, but in the left eye, the patient experienced no light perception (NLP). An exam revealed a dense relative afferent pupillary defect (RAPD) in the left eye, and intraocular pressure was normal in both eyes. The left eye had proptosis, ptosis, a complete loss of eye movement, and a frozen orbit with a loss of sensation on the left side of the face. The fundus examination revealed a pale, edematous retina with a cherry‐red spot in the left eye, indicating central retinal artery occlusion (CRAO). In contrast, the right eye was entirely normal.

**Figure 1:**
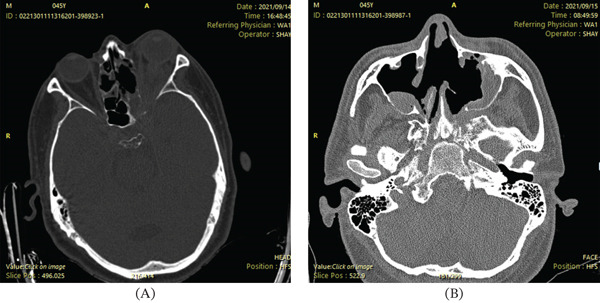
(A) Axial section of orbital compound tomography (CT) scan without contrast demonstrates intraorbital invasion in the left eye. (B) Axial section of paranasal sinuses CT scan shows evidence of paranasal sinuses debridement and collection in both maxillary sinuses.

An orbital computed tomography (CT) scan was performed due to orbital apex syndrome caused by ROCM, revealing that the fungus had invaded the orbital area (Figure 1). As a result, a three‐time retrobulbar amphotericin B liposomal injection was given, and the ROCM responded positively to the treatment. The patient was expected to be discharged.

**Figure 2:**
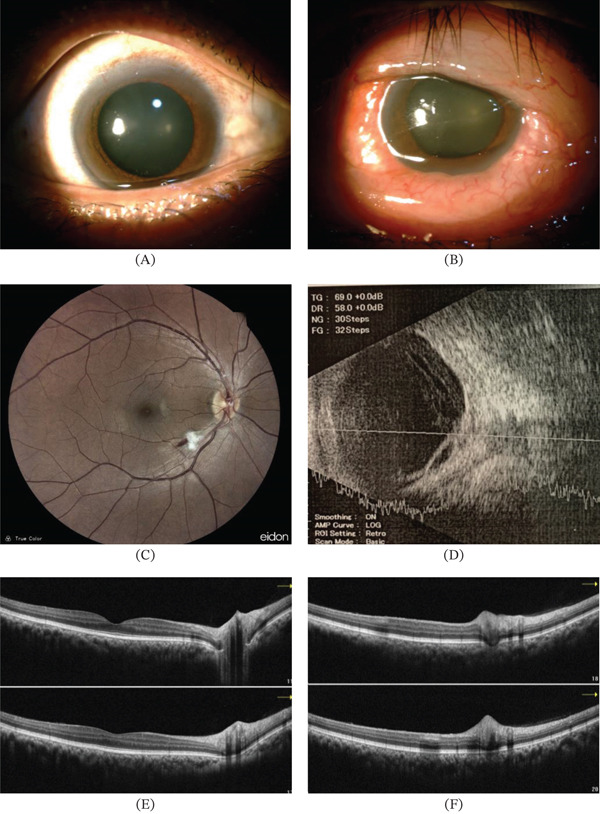
A slit lamp photograph of (A) the right eye shows no pathologic finding; hence, (B) the left eye shows severe chemosis. (C) Fundus photograph of the right eye (Day 37) shows the white retinal patch with hemorrhage in the posterior pole and near the inferotemporal arcade. (D) Ultrasound imaging of the left eye shows vitritis and retinal detachment. (E) Macula optical coherence tomography (OCT) was normal. (F) OCT of the retinal lesion shows hyperintensity of the inner and outer retina with a posterior shadow.

The patient reported a paracentral scotoma in the right eye 37 days after presenting with orbital involvement. UCVA was 20/20 in the right eye and NLP in the left, which showed prominent vitritis and a nonvisible fundus. A white retinitis‐like patch with adjacent preretinal hemorrhage was observed in the right eye (Figure 2). With a presumed bilateral endogenous fungal endophthalmitis diagnosis, we performed vitreous sampling from the left eye through a 27‐gauge vitrectomy port with intravitreal amphotericin B injection. Then, the smear report confirmed the presence of septated hypha in the vitreous sample, and evidence was suggestive of *Aspergillus*. Later, the culture revealed *Aspergillus fumigatus* as the pathogen. At that time, we decided to add oral posaconazole (40 mg/mL oral suspension) to the treatment regimen alongside continuing intravenous amphotericin B.

After 46 days in the hospital, he was discharged due to regression of ROCM, with negative biopsies from the nasal cavity and sinuses. During his stay, he was given an intravitreal amphotericin B injection in the left eye. Once discharged, he was prescribed posaconazole 10 mL daily and followed up as an outpatient. Upon follow‐up, enlargement of a preretinal hemorrhage and new retinitis‐like patches in the posterior pole and near the supratemporal arcade of the right eye were noted, but vitritis was absent. It was decided that his condition should be monitored with the previous prescription.

**Figure 3:**
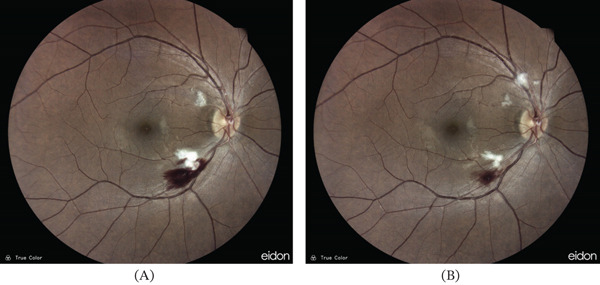
(A) Fundus photograph of the right eye (Day 46) shows an enlargement of the former epiretinal hemorrhage and retinal lesions and a new white retinal patch in the posterior pole and near the supratemporal arcade. (B) Fundus photograph of the right eye (Day 56) shows a reduced size and density of hemorrhage and cotton wool spots.

After 10 days of treatment with oral posaconazole, we observed a positive response with a reduction in the size and density of hemorrhage and retinitis‐like patches, as shown in Figure 3. Three months after starting the treatment, the inflammation in the left eye had subsided, and all the retinal lesions in the right eye had regressed. Unfortunately, the left eye lost its vision without any pain. The patient is currently under observation, and during 1 year of follow‐up, there has been no recurrence of the condition.

## 3. Discussion

This report describes a patient with COVID‐19‐associated ROCM who developed endogenous *Aspergillus* endophthalmitis in both eyes. The vitreous sample was positive for *Aspergillus fumigatus*, and multiple samples from nasal and perinasal sinuses were positive for mucormycosis. The patient, who was relatively young and had DM, received intensive dexamethasone treatment. This treatment may have led to an immunodeficiency state associated with COVID‐19, making the patient more susceptible to opportunistic pathogens such as *Mucor* and *Aspergillus*.

Mucormycosis and endogenous *Aspergillus* endophthalmitis are opportunistic infections that are associated with risk factors such as DM (especially ketoacidosis), prolonged and intensive corticosteroid use, hemochromatosis, HIV, neutropenia, malnutrition, and hematologic malignancies [3, 4]. These infections have been reported to occur more frequently in patients who have had COVID‐19 [1, 5].

Following COVID‐19, there has been an increase in fungal infection incidence, which is believed to result from the interaction between various factors. The decrease in T lymphocyte count can negatively impact the natural immune system. Additionally, there are similarities in angioinvasive processes between COVID‐19 infection and the mentioned pathogens. Finally, excessive corticosteroid use in some cases can further suppress the immune system [5–7].

There are only a few reports about ROCM with ipsilateral endogenous endophthalmitis [8–10] and one about contralateral endogenous endophthalmitis [11]. Bhansali et al., in a study on presentation and outcome of ROCM in 35 patients with DM, reported two cases of *Mucor* endophthalmitis [8]. Yohai et al. reported one case of *Mucor* endophthalmitis among 80 patients with ROCM. As far as we know, this report is the first case of ROCM associated with COVID‐19 that caused bilateral endogenous endophthalmitis. Ho et al. reported a patient who had unilateral ROCM with CRAO on the same side and choroiditis on the other side that progressed to endogenous fungal endophthalmitis. The patient received treatment with sinus debridement and systemic and intravitreal amphotericin B, which resolved the endophthalmitis. However, the ROCM eye remained blind due to CRAO [11]. Our case is interesting because a second pathogen was present alongside ROCM, which coincidentally caused bilateral endophthalmitis.

Managing ROCM involves a combination of antifungal therapy, surgical debridement, and controlling predisposing factors such as high blood sugar or discontinuing corticosteroids [1, 4]. The approved systemic antifungal agents for treating ROCM are amphotericin B, isavuconazole, and posaconazole. The retrobulbar injection of 3.5 mg/mL amphotericin B has also shown effectiveness in sight‐threatening ROCM [1]. Amphotericin B and posaconazole have been found to be effective in treating systemic *Aspergillus* infections [12]. We have also observed regression of the condition with the use of posaconazole. Despite losing vision in the left eye with NLP, the patient survived despite the recognized high mortality rate of mucormycosis infection [4]. The right eye is under observation, and hopefully, the retinitis lesions have finally regressed.

In conclusion, in light of the current COVID‐19 pandemic, it is imperative to recognize the potential complications that may arise during the administration of corticosteroids for treatment. Such complications may pave the way for the emergence of opportunistic infections, including but not limited to ROCM and *Aspergillus* endophthalmitis. Therefore, it is crucial to remain vigilant and take necessary precautions to mitigate the risk of these infections in COVID‐19 patients. Moreover, the possibility of multiple opportunistic pathogens coinciding should be considered in such cases. As such, healthcare professionals must maintain a high level of awareness and expertise to provide optimal patient care.

## Author Contributions

All the authors contributed significantly to this report, and all authors agree to be accountable for all aspects of the work.

## Funding

No funding was received for this manuscript.

## Disclosure

All authors read and approved the final manuscript.

## Ethics Statement

The authors have nothing to report.

## Consent

Consent for publication was acquired from the patient.

## Conflicts of Interest

The authors declare no conflicts of interest.

## Data Availability

The datasets used during the current study are available from the corresponding author upon reasonable request.
